# Kimura’s disease: a case report

**DOI:** 10.1186/s13256-024-04352-2

**Published:** 2024-02-06

**Authors:** Zablon Mesfin Anbessie

**Affiliations:** Bethzatha General Hospital, 57060 Addis Ababa, Ethiopia

**Keywords:** Subcutaneous nodules, Lymphadenopathy, Kimura’s disease

## Abstract

**Background:**

Kimura’s disease is a rare chronic inflammatory disorder of unknown etiology that is seen in people of Asian descent. It is characterized by head and neck subcutaneous nodules along with lymphadenopathy, which is usually solitary but can be generalized. It is diagnosed histopathologically by the proliferation of blood vessels and germinal centers in lymphoid follicles, along with variable degrees of fibrosis and extensive eosinophil infiltration. Its localized form is treated with surgical excision, while generalized lesions and those that do not respond to surgical excision can be managed with steroids or radiotherapy.

**Case:**

In this report, we present the first case of Kimura’s disease in the Ethiopian literature in a 40-year-old Ethiopian man that presented with generalized pruritic subcutaneous nodules and lymphadenopathy, which were effectively managed with a tapering course of prednisolone, and a relapse that showed good sustained response with slow steroid taper.

**Conclusion:**

We have demonstrated that, even though it is very rare in the African continent, Kimura’s disease is to be considered as a differential diagnosis for patients that present with subcutaneous nodules and lymphadenopathy. We also have demonstrated that relapses can be effectively managed with reinitiation of the same dose of steroids but with a very slow taper.

## Background

Kimura’s disease (KD) is a rare chronic inflammatory disorder of unknown etiology that involves the lymph nodes and subcutaneous tissue with a pathologic hallmark of angiolymphoid proliferation. Even though the etiology is not known, it is postulated that it may be caused by a dysregulated immune response or an atopic reaction to a persistent stimulus, such as an arthropod bite, virus, or neoplasm. It is speculated that genetics and sex hormones affect its development [1–3].

KD is usually seen in young Asian males between the ages of 20–40 years but is otherwise rare in other races. Among Asian countries, it is commonly reported in China, Japan, and Southeast Asia. The male-to-female ratio is 3:1. Ten cases of KD in black people have been described so far: six from the USA, two from Nigeria, one from Senegal, and one from Tunisia [4]. It usually presents with painless subcutaneous nodules in the head and neck with associated regional lymphadenopathy. It is solitary and unilateral in most patients. KD predisposes patients to allergic diseases such as asthma and chronic urticaria. On investigation, patients can have peripheral eosinophilia with increased serum IgE levels [1–5].

The gold standard for the diagnosis of KD is the pathologic examination of the involved tissue. Histological features can be classified as constant, frequent, and rare. Constant features include germinal center hyperplasia, postcapillary venular proliferation, preservation of nodal architecture, and infiltration by eosinophils. Frequent features include necrosis, abscess formation, IgE reticular deposition, vascular proliferation, and proteinaceous deposits in the germinal center with background sclerosis. A rare feature is germinal center transformation [1, 3].

The differential diagnosis for KD includes angiolymphoid hyperplasia, which is seen in Caucasians with superficial papules and an uncommon finding of lymphadenopathy, Hodgkin’s disease, and Kaposi’s sarcoma, which have characteristic histologic findings. Other less common illnesses include eosinophilic granuloma, Castleman’s disease, and drug-induced lymphadenopathy. The clinical course is generally benign, and it is self-limited in up to 25% of patients. Treatment is controversial, as there are no large-scale studies. The use of surgery along with steroids and radiotherapy is what is recommended by experts. Surgical excision is considered the gold standard for solitary lesions, as it reduces lumps with no risk of spreading the disease. Surgery with postoperative intervention is reasonable for patients with subcutaneous lesions, extensive lesions, and those without clear margins. Radiotherapy (XRT) is reserved for recurrent cases. Radiation doses ranging from 20–45 Gy have been reported to be successful. Chinese researchers have suggested that imatinib might be helpful in patients with positive platelet-derived growth factor receptor alpha (PDGFRA) positivity. The disease recurs in up to 25% of patients and recurrences are more common in the head and neck. [1, 3, 5]

## Case report

A 40-year-old Ethiopian male presented to our hospital complaining of painless swellings under the chin and back of the neck for 6 months. The swellings, after they appeared, were 3 months later, followed by itchy heaped-up swellings over the forearms, thighs, legs, and finally the face. These swellings increased in itchiness and size during the daytime and sunlight exposure. Two months before the presentation, he developed new swellings that he noticed over his axillary and inguinal areas. He was treated with oral loratadine and topical steroids as a case of nodular urticaria with no significant improvement. Two weeks before his presentation, he developed worsened fatigue, loss of appetite, and epigastric pain. Three days before his presentation, he developed a high-grade fever, headache, chills, rigors, and worsened epigastric pain.

**Fig. 1:**
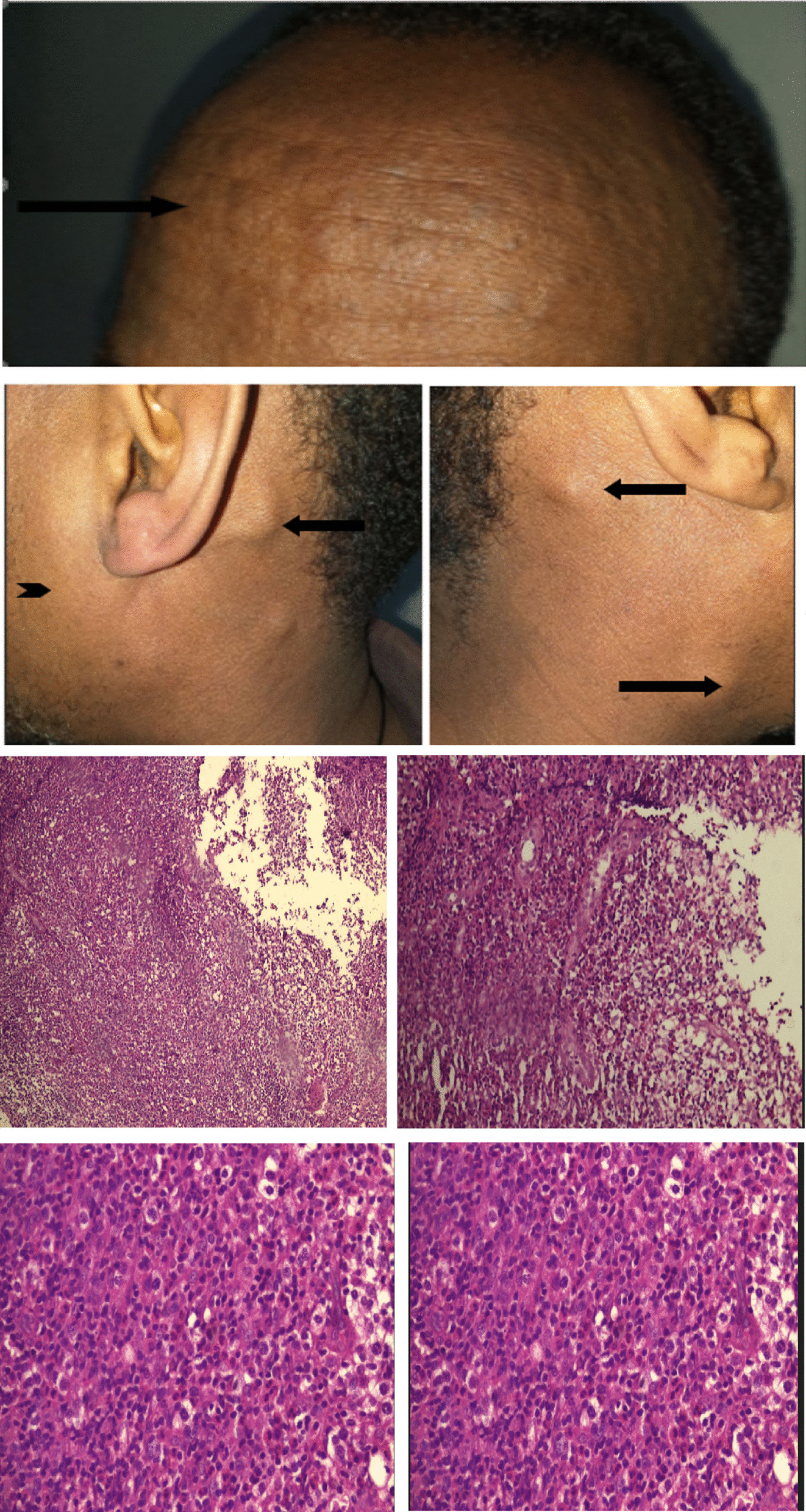
Skin nodules and hyperpigmentation with lymphadenopathy (arrows), parotid gland enlargement (arrowhead), and hematoxylin and eosin-stained lymph node biopsy showing florid hyperplasia and vascularization of follicles along with parafollicular eosinophil infiltration, vascular hyperplasia, and background fibrosis

On examination, his pulse rate was 95 beats per minute, his respiratory rate was 20 breaths per minute, and he had a temperature of 36^0C^ and a saturation of 94% on room air. He had injected conjunctiva with a puffy face. He had multiple nontender firm lymph nodes of different sizes ranging from 2 to 4 cm over the submandibular, anterior and posterior cervical, axillary, posterior auricular, and inguinal areas bilaterally. He had no organomegaly. Skin evaluation that was done by a dermatologist showed generalized urticarial papules involving the face, neck, and upper extremities, with macular erythematous rash involving the trunk, and eczematous plaques and excoriation marks over the extremities. (Fig. 1).

His investigations showed normal liver function, renal function, coagulation, and lipid panel. His viral hepatitis and human immunodeficiency virus (HIV) tests were negative. His VDRL was positive, but his TPHA was negative. He had a white cell count of 4500 cells/mm^3^ with 65% neutrophils, 19% lymphocytes, and 9% eosinophils. He had a hemoglobin of 13.7 g/dl and a platelet count of 284,000 cells/mm^3^. His fasting blood sugar on two occasions was 86 and 98 mg/dl.

His lymph node biopsy showed florid hyperplasia and vascularization of the germinal centers and parafollicular areas with proliferation and infiltration by eosinophils with fibrotic changes. His serum platelet-derived growth factor receptor alpha (PDGFRA) assay was negative.

With a diagnosis of PDGFRA-negative Kimura’s disease, he was started on a tapering dose of prednisolone 30 mg/d. After 2 weeks of therapy, all symptoms including lymph node swelling and skin nodules resolved. Four months later, he was taken off prednisolone and had a similar recurrence 6 months after drug discontinuation that required reinitiation of prednisolone. After reinitiation of the same dose, a similar fast taper was attempted, which caused a recurrence of symptoms; so, a slow taper with 5 mg every month was attempted with a good response, with the patient being symptom-free while on 5 mg of prednisolone a year later.

## Discussion

We have shown in our case report that, even though Kimura’s disease is a rare disease that is even rarer in the African population, it must be among the differential diagnoses for head and neck nodules with lymphadenopathy. We have also demonstrated that the disease can be diagnosed with histopathology and can be effectively treated with oral steroids even though at a high risk of recurrence.

In a retrospective review of 24 patients with Kimura’s disease from China, it was demonstrated that the median age of patients was 44.5 years (5–65 years), with 88% being above 35 years. A total of 83 percent of the patients were males with nodule and lymph node sizes ranging from 0.6 to 7 cm. The patients were symptomatic from 2 months to 17 years before diagnosis, with the most common findings on the diagnosis being: predominant solitary and unilateral pruritic subcutaneous nodules, parotid gland swelling, and hyperpigmented coarse skin. A total of 87% of the patients had peripheral eosinophilia of more than 5%. Reports of Kimura disease from Egypt, Nigeria, and Tunisia all reported patients with age and clinical presentations similar to the case series above. Our patient had all findings described in the literature, but he was different in that his lymphadenopathy and skin involvement was more generalized, which is a less common presentation of Kimura’s disease. [1–5]

In the Chinese review, the most common histopathologic finding was hyperplasia of the germinal centers and vessels in the germinal centers of lymphoid follicles. There was universal fibrosis and infiltration of the lymphoid follicles with eosinophils. Our patient fulfills all histopathologic criteria and features that are seen in these Chinese patients. [1]

The Chinese review classified the patients for management purposes as patients with localized disease who can be given surgical excision with steroids or radiotherapy and those with a generalized disease that can only be given steroids. The Egyptian case report administered a high dose of prednisolone at 60 mg/day for the co-occurrence of nephrotic syndrome, while the Nigerian report administered the same high dose of prednisolone for patients with no comorbidity. The Nigerian report showed that the recurrence was higher after 15 days of treatment followed by withdrawal of medication. Our patient had a generalized disease and was not a candidate for imatinib, so, he was given 30 mg/day of prednisolone, which was tapered off. He had a complete remission in 15 days, but he relapsed 6 months later. After his relapse, he was restarted on 30 mg of prednisolone, and the dose was tapered by 5 mg every month with his current dose being 5 mg per day with no symptoms.. [1, 2, 4, 5]

Despite being very rare, and likely underreported in developing countries due to the indolent nature of the disease and the lack of access to care, Kimura’s disease should be kept in mind while evaluating a black patient that presents with subcutaneous nodules and lymphadenopathy, where infectious and allergic diseases would take more precedence.

Our report is limited in the fact that we were unable to perform further immunohistochemistry tests but had the opportunity to follow the patient for more than 1 year with good remission being possible after a slow taper of prednisolone.

## Conclusion

This report has attempted to provide baseline evidence for Ethiopia and Africa regarding the diagnosis and nonoperative management of Kimura’s disease. It also highlighted the difference in the presentation of this African patient from the patients reported from Asian countries. It has also shown that the disease relapses if steroids are tapered early and quickly.

## Data Availability

All have been included in the manuscript.

## Abbreviations

Gy: Gray
HIV: Human immunodeficiency virus
KD: Kimura’s disease
PDGFRA: Platelet-derived growth factor receptor alpha
XRT: Radiotherapy
